# A new species of *Elasmia* Möschler from New Mexico and Texas, and a new subspecies of *Elasmia mandela* (Druce) from Texas and Oklahoma (Lepidoptera, Notodontidae, Nystaleinae)

**DOI:** 10.3897/zookeys.149.1519

**Published:** 2011-11-24

**Authors:** Eric H. Metzler, Edward C. Knudson

**Affiliations:** 1 Adjunct Curator of Lepidoptera, Michigan State University, P.O. Box 45, Alamogordo, NM 88311-0045 USA; 2 Texas Lepidoptera Survey, 8517 Burkhart Rd., Houston, TX 77055-7517 USA

**Keywords:** Lepidoptera, Notodontidae, Nystaleinae, Arizona, Oklahoma, New Mexico, Texas, Kansas, *Hippia*, *Elasmia*, Carlsbad Caverns National Park, Santa Ana National Wildlife Refuge

## Abstract

*Hippia packardii* (Morrison) and *Hippia insularis* (Grote) are moved to the genus *Elasmia* Möschler as **comb. n.** *Elasmia cave* Metzler*,* **sp. n.** is described from New Mexico and Texas, and *Elasmia mandela santaana* Metzler & Knudson*,* **ssp. n.** is described from Texas and Oklahoma. A key to the species of *Elasmia* of southwestern U.S. is provided. Adult male and female moths of *Elasmia* from southwestern U.S. and their genitalia are illustrated.

## Introduction

Lafontaine and Schmidt (2010) listed two species of *Hippia* Möschler, 1878 (Notodontidae: Nystaleinae) for North America north of Mexico: *Hippia packardii* (Morrison, 1875), described from Texas; and *Hippia insularis* (Grote, 1866), described from Cuba. They listed no species of *Elasmia* Möschler, 1886 (Notodontidae: Nystaleinae). Our investigations show that *Hippia packardii* and *Hippia insularis* belong in the genus *Elasmia*, and that *Hippia insularis*, reported from Texas (Knudson and Bordelon 1999), is an error. United States specimens thought to be *Hippia insularis* instead represent an undescribed species described here as *Elasmia cave*. Our investigations further show an undescribed subspecies of *Elasmia mandela* (Druce, 1887) from the United States. *Elasmia mandela santaana* is described here from Texas and Oklahoma.

## Methods

Adult moths were collected in U.S.D.A. type black-light traps and at black light and sheet as described in Covell (1984).

Genitalia were examined following procedures outlined in Clarke (1941), Hardwick (1950), Lafontaine (2004), and Pogue (2002). Abdomens were removed, wetted in 95% ethyl alcohol, and soaked in 10% KOH for 1.5 hours at 50˚C. Genitalia were dissected in 5% ethyl alcohol, stained with Safranin O in ethyl alcohol and Chlorozol Black in water, dehydrated in 100% ethyl alcohol, cleared in oil of cloves, rinsed in xylene, and slide mounted in Canada balsam.

The aedeagus of species of *Elasmia* is held firmly in place by membranes within the genital capsule, and the aedeagus is nearly always broken into two pieces during the process of removal. The anterior portion is short and abruptly flared out. The posterior portion with the everted vesica is illustrated in this paper (figs 13, 16, 19).

Wing pattern terminology came from (Lafontaine (1987, 2004) and Mikkola et al. (2009). Morphological structure terminology came from Common (1990) and Forbes (1954), Genital structure terminology came from (Lafontaine (1987, 2004), Franclemont (1946), Forbes (1954), Klots (1970), Miller (1991), and Weller (1995). Forewing lengths, from the base to the apex excluding fringe, were measured to the nearest mm, using a stereo-microscope. Nearly all specimens from New Mexico were collected as part of a long-term faunal study of Lepidoptera at Carlsbad Caverns National Park.

Specimens of Lepidoptera from this study are deposited in the following collections:

AMNH American Museum of Natural History, New York, New York

BMNH Natural History Museum, London, England

CMNH Carnegie Museum of Natural History, Pittsburgh, Pennsylvania

CUIC Cornell University, Ithaca, New York

EHM Eric H. Metzler, Alamogordo, New Mexico, for subsequent transfer to MSU

JBS J. Bolling Sullivan, Beaufort, North Carolina

JBW J. Bruce Walsh, Tucson, Arizona

MSU Albert J. Cook Arthropod Research Collection, Department of Entomology, Michigan State University, East Lansing, Michigan

MCZ Museum of Comparative Zoology, Harvard University, Cambridge, Massachusetts

ORU Oral Roberts University, Tulsa, Oklahoma

OSU Oklahoma State University, Stillwater, Oklahoma

TAM Texas A&M University, College Station, Texas

TLSRC Edward C. Knudson, Texas Lepidoptera Survey, Houston, Texas

UFL McGuire Center for Lepidoptera and Biodiversity, University of Florida, Gainesville, Florida

UNM Museum of Southwestern Biology, University of New Mexico, Albuquerque, New Mexico

USNM United States Museum of Natural History (Smithsonian Institution) Washington, DC

## Results

### Key to the species of *Elasmia* in Arizona, Kansas, New Mexico, Oklahoma, and Texas

**Table:** 

1	Forewing gray and/or blue gray, sides of uncus convex, evenly curved (Fig. 11), forewing length = 13–16 mm	*Elasmia packardii*
–	Forewing brown or gray, sides of uncus flared outward (Figs 14, 17), forewing length = 14–18 mm	2
2	Forewing brown gray (Fig. 5), apex of costulae swollen and bent	*Elasmia cave*
–	Forewing gray brown (Fig. 9), apex of costulae straight and not bent	*Elasmia mandela santaana*

In south central Texas, larvae of the genus *Elasmia* (not identified to species)feed on *Ungnadia speciosa* Endl. (Mexican buckeye) and *Sapindus saponaria var. drummondii* (Hook. & Arn.) L. Benson (soapberry tree) (both Sapindaceae) (Val Bugh pers. comm. 2010).

## Systematics

### Hippia

Möschler, 1878

#### Discussion.

We examined the illustration of the type and the male and female genitalia of *Hippia mumetes* (Cramer, [1775]), the type species of *Hippia* Möschler (1878). Those examinations show that the North American species, placed in *Hippia*, are not congeneric with *Hippia mumetes*.

### Elasmia

Möschler, 1886

#### Discussion.

We examined the illustration of the type of *Elasmia lignosa* Möschler, 1886, the type species of *Elasmia*. The North American species, previously placed in *Hippia*, are determined to be congeneric with *Elasmia*

### Elasmia insularis

(Grote, 1866) comb. n.

#### Discussion.

The adult and genitalia of *Elasmia insularis* were illustrated in Torre and Alayo (1959). We examined male specimens, and their genitalia, of *Elasmia insularis* from Cuba. *Elasmia insularis* is not known to occur in Florida (Heppner 2003), and it is doubtful that it occurs in the U.S. Inclusion of *Elasmia insularis* in Lafontaine and Schmidt (2010) was based on erroneous reports from Texas (Knudson and Bordelon 1999).

### Elasmia packardii

(Morrison, 1875) comb. n.

http://species-id.net/wiki/Elasmia_packardii

Figs 1–4 11–13 20, 23 26

#### Description.

Overall color light gray blue to gray with obscure transverse forewing markings, sometimes showing slight brownish shadings over reniform spot and in postmedial and subterminal areas. Males and females similar in appearance; male antenna narrowly bipectinate in basal ¾, with dense setae on ventral surface. Female antenna filiform for entire length, with sparse setae. Apex of forewing marked with a diagonal white and dark shade. Forewing length in males 12–15 mm (mean = 14 mm, n = 72), and in females 13–16 mm (mean = 14 mm, n = 25). Male genitalia (Figs 11–13) distinguished by a helmet-shaped uncus with gradually widening sides. Female genitalia (Fig. 20) with membranous papilla anales partially hidden from view. Ductus bursae broad and short, dorso-ventrally compressed; corpus bursae, round in profile, with a single shark tooth-shaped signum, also with a heavily sclerotized, perpendicular, thumb-like projection ventrally and a sclerotized finger-like pocket appressed to corpus bursae dorsally. Deciduous cornuti from male vesica may be found in corpus bursae.

#### Remarks.

Morrison (1875) described *Elasmia packardii* from Waco, Bosque County, Texas (Fig. 2) based on a single female specimen (Fig. 1). Adults are on the wing from April through early October.

#### Distribution and Biology.

*Elasmia packardii* occurs in Texas, Arizona, New Mexico, Oklahoma, and Kansas (Fig. 26); it is common at Carlsbad Caverns National Park. Its distribution in Mexico is unknown. The larvae feed on *Ungnadia speciosa* Endl. (Mexican buckeye) (R.O. Kendall specimens in TAM) and *Sapindus saponaria* var. *drummondii* (Hook. & Arn.) L. Benson (soapberry tree) (both Sapindaceae) (R.O. Kendall specimens in AMNH and TAM).

**Figure:**
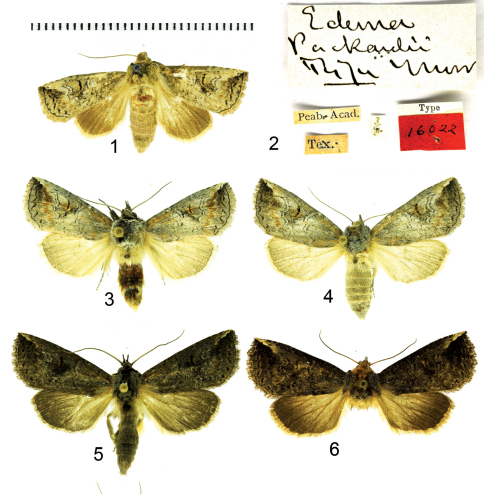
**Figures 1–6.** *Elasmia*adults. **1** *E*. *packardii* female holotype **2** *E*. *packardii* holotype labels **3** *Elasmia packardii* male **4** *Elasmia packardii* female **5** *Elasmia cave* male holotype **6** *Elasmia cave* female paratype.

**Figure:**
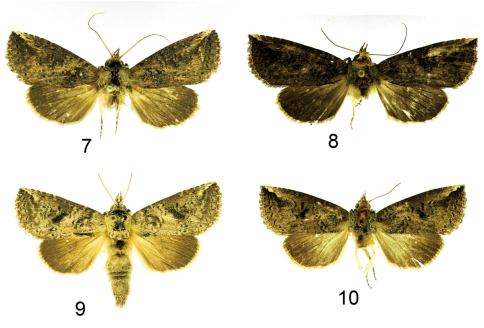
**Figures 7–10.** *Elasmia*adults. **7** *Elasmia mandela* male **8** *Elasmia mandela* female **9** *Elasmia mandela santaana* male holotype **10** *Elasmia mandela santaana* female paratype.

**Figure:**
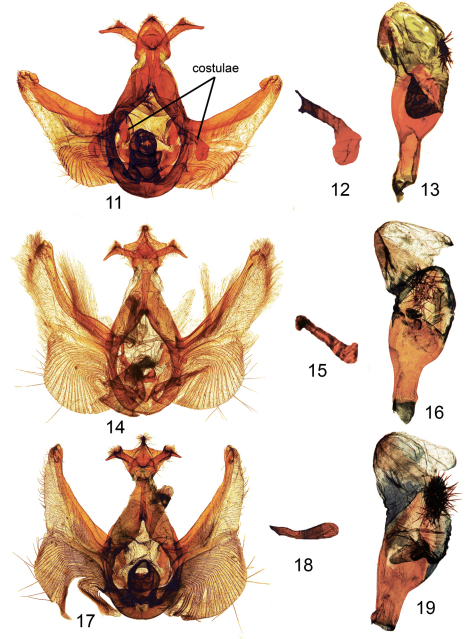
**Figures 11–19.** *Elasmia*male genitalia. **11** *Elasmia packardii* male genitalia genital capsule slide E.H.M. 343 **12** *Elasmia packardii* male genitalia detail of terminus of costulae slide E.H.M. 343 **13** *Elasmia packardii* male genitalia aedeagus slide E.H.M. 343 **14** *Elasmia cave* male genitalia paratype genital capsule slide E.H.M. 355 **15 ***Elasmia cave* male genitalia detail of terminus of costulae slide E.H.M. 355 **16** *Elasmia cave* male genitalia paratype aedeagus slide E.H.M. 355 **17** *Elasmia mandela santaana* male genitalia paratype genital capsule slide E.H.M. 359 **18** *Elasmia mandela santaana* male genitalia detail of terminus of costulae slide E.H.M. 359 **19** *Elasmia mandela santaana* male genitalia paratype aedeagus slide E.H.M. 359.

### Elasmia cave

Metzler sp. n.

urn:lsid:zoobank.org:act:93E887C7-757F-4EC6-B5B5-5E4012D9822A

http://species-id.net/wiki/Elasmia_cave

Figs 5–6 14–16 21, 24 27

#### Type material.

Holotype male: “USA: NM: Eddy Co. Carlsbad Caverns N[ational] P[ark], riparian habitat, 32°06.566' N 104°28.257' W, 29 August 2006, Eric H. Metzler, CCNP4, uv trp Accsn #: CAVE - 02263", “HOLOTYPE USNM *Elasmia cave* Metzler” [red handwritten label] (USNM). Paratypes: 19 males; 14 females: NEW MEXICO: USA: NM: Eddy Co. Carlsbad Caverns NP, arroyo habitat 32°05.98'N 104°33.57'W, 5 September 2010, Eric H. Metzler, CCNP2 uv trp Accssn #: CAVE - 02263. TEXAS: Alpine, Tex., 1–7 May 1926, 8–14 May 1926, 1–7 July 1926, 8–14 July 1926, 15–21 July 1926, 1–7 Aug. 1926, 15–21 Aug. 1926, O.C. Poling, Coll[ector]. Barnes Collection (USNM), Texas, Uvalde Co. Concan, 12-V-90, leg. E.C. Knudson. 14-X-93, Concan, Uvalde Co., TX, Coll C. Bordelon. TX: Brewster Co., Big Bend N.P., Green Gulch/5400' 5–7-V-97/ECK. Big Bend, Tex. Brewster Co., 6–7000 ft., Poling, F. Johnson donor, 8-1-26. USA: Texas: Jeff Davis Co. Davis Mountains, Limpia Canyon, elev: 4920', 30°30.0'N 103°52.5'W, 8 August 1991, Eric H. Metzler. TEXAS: Jeff Davis Co., 25-VI-81, Davis Mt. St. Pk., Jeff Davis Co. TX: Ft. Davis, 3-x-94, leg. E. Knudson. 24 Aug 1995. Jeff Davis Co. Texas, 5-V-78, Kokernaut Creek, leg. E.C. Knudson. Jeff Davis Co., TX, Ft. Davis, 10,11-IX-10 Bordelon & Knudson coll. Jeff Davis Co., TX, Ft. Davis, 24–26-V-07 Bordelon & Knudson coll. TX: Culberson Co., Guadalupe Mts. N.P., Lamar Cyn., Coll. C. Bordelon. TEXAS: Culberson Co., Guadalupe Mts. N.P., Pine Spring, 6–8-IX-91, leg. E.C. Knudson. Green Gulch 5400' Big Bend Natl. Park Brewster Co., Texas 4 May 1972 J. G. Franclemont ♂ Genitalia slide 6419 J. G. Franclemont. Alpine, Brewster Co. Texas 15–21 Aug. 1926 O.C. Poling ♂ Genitalia slide 2535 J. G. Franclemont. (EHM, MSU, CUC, TLSRC, USNM).

#### Etymology.

CAVE is the acronym, used by the U.S. National Park Service, for Carlsbad Caverns National Park. The specific name of this species, *cave*, treated as a noun in apposition, refers to the type locality, Carlsbad Caverns National Park.

#### Diagnosis.

*Elasmia cave* is a dark brown-gray moth with obscure transverse markings. *Elasmia cave* looks like brown example of *Elasmia mandela*; *Elasmia mandela* is dark gray brown. The brown color of the imago and its genitalia will separate *Elasmia cave* from *Elasmia packardii*, whichis gray blue to gray. The uncus of *Elasmia packardii* (Fig. 11), narrow at its apex, gradually widens with evenly curved sides. The uncus of *Elasmia cave* (Fig. 14) is wide, like a manta ray, and narrows immediately before the apex. The distal end of the costulae of *Elasmia insularis*, n=3,are narrow, straight or slightly sinuous, and without bend or swelling apically (illustrated by Torre and Alayo 1959); the costulae of *Elasmia mandela*, n=3, are nearly identical to *Elasmia insularis*. In comparison to *Elasmia insularis* and *Elasmia mandela*, the costulae of *Elasmia cave* (Fig. 15) are broader, and they are abruptly bent upward and swollen at the distal end.

#### Description.

Adult male (Fig. 5): *Head*: smoky gray, scales strap-like, erect, a fuscous line between eyes below antennae. Labial palpus erect, extending upward to slightly beyond base of antenna, smoky brown gray with a fuscous lateral stripe, extends to slightly beyond base of antenna, ventral scales on 1st and 2nd segments long, not shaggy, 3rd segment closely scaled. Haustellum coiled between labial palpi. Antenna narrowly bipectinate for basal 3/4, each ramus tipped with long setae, apical 1/4 ciliate with short setae, dorsal surface alternating fuscous and smoky, closely scaled, ventral surface naked, brown. *Thorax*: collar black, sometimes preceded by brown, dorsum smoky brown gray, longitudinal narrow black lines anteriorly, posteriorly, and laterally, tegula smoky brown gray, scales strap-like; underside smoky dark gray brown, laterally smoky, scales erect long hair-like or narrowly forked. Legs: dark smoky gray brown, closely scaled, except lateral margin with shaggy scales, tarsomere apex yellow. Forewing: length 14–18 mm, mean 16 mm, n = 16; dorsal surface ground color smoky gray brown, sometimes slightly hoary; antemedial line obscure, pale basally, black mesally, sinuous; postmedial line vague, sinuous, black basally outer element pale; subterminal line a series of fuscous bars; terminal line narrow, black; orbicular spot absent or vaguely pale; reniform spot inconspicuous, dark with pale outline; costa brown except white shade at apex; subreniform spot fuscous, contrasting; dark line from apex running obliquely toward reniform spot; fringe smoky gray. Ventral surface: smoky dark gray black, apical markings similar to dorsal surface, fringe concolorous. Hindwing dorsal surface: dark smoky gray, slightly paler basally, markings absent, fringe pale smoky. Ventral surface: apex to tornus dark smoky gray, tornus pale gray along inner margin, base pale gray, markings absent, fringe pale gray. *Abdomen*: dorsum smoky gray, with fuscous tufts on first and second segments, elsewhere closely scaled, underside pale smoky gray. *Genitalia* (Fig. 14): Uncus broad, flattened, setose, apex bluntly pointed, dorsally with narrow ridge, ventrally with two short cornutus-like spines; socii broad, setose, bent at approximately 90°, with one ear-like dorsal projection; tegumen flattened; saccus short, broadly U-shaped; juxta shield shaped, dorsal margin a half circular cutout; diaphragma bearing two sclerotized processes (costulae) near bases of valvae costa, bent at 90°, bent and swollen club-like apically (Fig. 15), valve setose, dorsally sclerotized, ventrally membranous, Barth’s Organ large, with many chevron-shaped parallel pleats, cucullus not well differentiated, with three narrow, curved ridges, corona with weak, mesally-directed curved setae. Aedeagus (Fig. 16) straight, anterior end abruptly flared out, posteriorly flattened, spoon-shaped; vesica lightly sclerotized, subbasal diverticulum with a nipple-shaped cornutus; a patch of deciduous (may be dislodged during mating) stellate (like a starfish) spicule-shaped cornuti; basal diverticula lightly sclerotized, with two finger-like subbasal diverticulae.

Adult female (Fig. 6). Similar to male except; antenna filiform, without long setae, top of head yellow to orange, collar to disc of thorax yellow to orange. Forewing length 15–18 mm, mean 17 mm, n = 11. *Genitalia* (Fig. 21). Papilla anales membranous, setose, partially hidden from view between sclerotized extensions of ninth abdominal segment. Posterior apophyses slender. Anterior apophyses slender. Ductus bursae short, broad. Corpus bursae round, with a single shark tooth-shaped signum; sclerotized ventral wall forming a thumb-like extension with bulbous terminus; sclerotized dorsal wall with a pock-marked, finger-like extension appressed to surface of corpus bursae.

#### Remarks.

*Elasmia cave* was mistakenly identified in the U.S. as *Elasmia insularis*. The costulae of the male genitalia, figured in (Torre and Alayo 1959) from Cuba and noted in the diagnosis, separate the species. *Elasmia cave* is placed in the genus *Elasmia* Möschler, 1886, because the imago is closely similar to *Elasmia lignosa*, and the male genitalia are closely similar to those of *Elasmia mandela*.

#### Distribution and biology.

*Elasmia cave* occurs in the U.S. in New Mexico and Texas; its distribution in Mexico is not known. Three specimens were collected in riparian habitats in Texas and New Mexico. Two specimens from Alpine, Texas and one from Big Bend, Texas, leg. Poling, have additional handwritten labels that say “Buckeye” or “bred Buckeye” respectively. The type locality was selected because it will be protected by the U.S. National Park Service into perpetuity.

**Figure:**
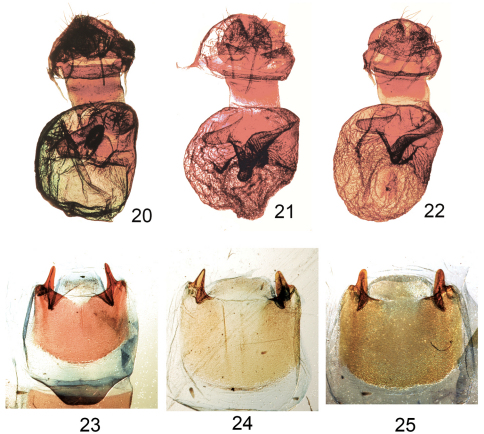
**Figures 20–25.** *Elasmia*female genitalia and male eighth sternites. **20** *Elasmia packardii* female genitalia slide E.H.M. 347 **21** *Elasmia cave* female genitalia paratype slide E.H.M. 409 **22** *Elasmia mandela santaana* female genitalia paratype slide E.H.M. 410 **23** Eighth sternite of male of *Elasmia packardii* slide E.H.M. 343 **24** Eighth sternite of male of *Elasmia cave* paratype slide E.H.M. 355 **25** Eighth sternite of male of *Elasmia mandela santaana* paratype slide E.H.M. 359.

### Elasmia mandela

(Druce, 1887)

http://species-id.net/wiki/Elasmia_mandela

Figs 7, 8

#### Description.

Overall color dark gray brown with obscure transverse forewing markings. Males and females similar in appearance. Male antenna narrowly bipectinate in basal ¾, with dense setae on ventral surface. Female antenna filiform for entire length, with sparse setae. Apex of forewing with a diagonal white mark. Reniform spot outlined with pale-orange scales, not contrasting. Forewing length in males 17.0–18.0 mm (mean = 17.2 mm, n = 5), and in females 18.0–20.0 mm (mean = 19.3 mm, n = 7). Male genitalia distinguished by uncus with abruptly widening sides, like a manta ray, and robust saccular area (Barth’s Organ). Female genitalia with membranous papilla anales that are partially hidden from view. Ductus bursae broad and short, dorso-ventrally compressed; corpus bursae round in profile, with a single shark tooth shaped signum, also with a heavily-sclerotized, perpendicular, thumb-like projection ventrally and a sclerotized finger-like pocket appressed to corpus bursae dorsally.

#### Remarks.

Druce (1887) described *Elasmia mandela* from Presidio, Mexico, based on a single female specimen. We examined a photograph of the type and it’s genitalia. We also examined specimens from Vera Cruz and Yucatan, Mexico (AMNH), and from Costa Rica (JBS).

#### Distribution and biology.

*Elasmia mandela* occurs in Mexico and Costa Rica. Its distribution in other Central American countries is unknown. The larval hosts in Costa Rica are one species of Rhamnaceae and 22 species of Sapindaceae (Janzen and Hallwachs 2009).

### Elasmia mandela santaana

Metzler & Knudson subsp. n.

urn:lsid:zoobank.org:act:9B30138B-9B92-4B81-9797-82B59ABE417F

http://species-id.net/wiki/Elasmia_mandela_santaana

Figs 9, 10 17–19 22, 25 28

#### Type material.

Holotype male: “Hidalgo Co. Texas 31-X-83 Santa Ana Refuge leg. E.C. Knudson” “HOLOTYPE USNM *Elasmia mandela santaana* Metzler & Knudson” [red handwritten label] (USNM). Paratypes: 13 males; 10 females: TEXAS: Harris Co: TX. Houston, Leg. E.C. Knudson, 9-VIII-75. Hidalgo Co. TX. Santa Ana NWR, 6-IX-92, leg. E.C. Knudson. Texas: Uvalde Co. Concan, 15-V-10, B/K. Tarrant Co. Texas Benbrook, 30-IV-78, leg. E.C. Knudson. Terrel Co. Tex. Sanderson, 25-IV-81, leg. E.C. Knudson. Kerrville. Texas, Barnes Collection. Kerrville, Texas. VIII 1904. Kerrville, TX. H. Lacy Collector. Kerrville, 4-23-08, TX. F.C. Pratt Collector. Texas, San Patricio Co. Welder Wildlife Refuge near Sinton, Texas, 14-16-VI-85, leg. E.C. Knudson. Hidalgo Co., TX, Bentsen State Park, 6-VIII-94, E. Knudson coll. Montgomery Co. Tex. Sawdust Rd. & I.S. 45, leg. E.C. Knudson, 20-VI-75. Brownsville, Tex III-10-29, F.H. Benjamin collr, Barnes Collection. Alpine, TX. 8–14 May, 8–14 July, 15–21 Aug. 1926, O.C. Poling, Coll[ector]. TEXAS: Smith Cany., Guadalupe Mountains, Culberson Co., 5750’ May 22, 1973, Douglas C. Ferguson. OK: Caddo Co. Methodist Youth Camp 1 October 1994 J.M. Nelson Coll. OK: Tulsa Co. Sand Springs 145^th^ & W. 19^th^ St. Aug 27 - Sept 1, 2008 J.F. Fisher, Collr. at black light. OK: Tulsa Co. Sand Springs 145^th^ & W. 19^th^ St. September 12, 2008 J.F. Fisher, Collr. at black light. (TLSRC, ORU, USNM).

#### Etymology.

The name of this subspecies, *santaana*,refers to its type locality, Santa Ana National Wildlife Refuge in Texas. The name is treated as a noun in apposition.

#### Diagnosis.

*Elasmia mandela santaana* is gray overall. The adult resembles a large example of *Elasmia packardii*; *Elasmia mandela santaana* has a contrasting dark scale patch in the reniform/subreniform area. *Elasmia mandela santaana* (mean forewing length = 16 mm) is larger than *Elasmia packardii* (mean forewing length = 14 mm) and *Elasmia mandela santaana*’s Barth’s Organ is relatively larger. The lateral margin of the uncus in *Elasmia packardii* has a slight shoulder immediately below the apex, whereas in *Elasmia mandela santaana* the lateral margin of the uncus is flared outward. *Elasmia mandela santaana* is a gray moth, and *Elasmia cave* is a brown moth. The male and female genitalia of *Elasmia mandela santaana* are similar to those of *Elasmia cave*. The costulae of *Elasmia cave* are abruptly bent and swollen apically (Fig. 15); the costulae of *Elasmia mandela santaana* may be slightly swollen but not bent apically (Fig. 18).

#### Description.

Adult male (Fig. 9): *Head*: smoky gray, scales strap-like, erect, a vague fuscous shade between eyes below antennae, a vague fuscous shade behind antennae. Labial palpus erect, extending to base of antenna, smoky brown gray with two dark-brown lateral lines, ventral scales on 1st and 2nd segments long, not shaggy, 3rd segment closely scaled. Haustellum coiled between labial palpi. Antenna narrowly bipectinate for basal 3/4, each ramus tipped with long setae, apical 1/4 ciliate, with short setae, dorsal surface smoky, closely scaled, ventral surface naked. *Thorax*: pale brown behind head; collar narrow, black, dorsum smoky with blackish brown-tipped scales on disc; tegula pale smoky, edged with black scales, scales strap-like; underside dark smoky gray with pale-tipped scales, smoky laterally, hair-like. Legs: smoky dark gray brown, closely scaled, each segment and each tarsomere apex ringed with pale. Forewing: length 15–17 mm, mean 16 mm, n = 7. Dorsal surface ground color smoky gray; basal line pale at costa, finely lined with black; antemedial line pale, sinuous, finely lined with black; postmedial line sinuous, pale, finely lined with black; subterminal line a series of fuscous black bars; terminal line a fine fuscous line; orbicular spot inconspicuous; reniform spot a black bar outlined with pale, dark blackish shade in lower part; subreniform spot contrasting black and fuscous; costa apex pale gray tan to white; dark line with dark shade from apex oblique to subreniform spot. Ventral surface smoky; terminal line black; fringe smoky. Hind wing. Dorsal surface ground color smoky gray, darker distally; fringe pale. Ventral surface ground color smoky, with scattered fuscous scales; fringe smoky. *Abdomen*: smoky, basal tuft blackish, underside smoky. *Genitalia* (Fig. 17): Uncus flattened, flared outward laterally, with narrow shoulders immediately below apex, apex setose, pointed; socii large, setose, bent at approximately 90°, each arm with an ear-like ridge; tegumen flattened; saccus U-shaped, short; juxta shield shaped, dorsal margin a half circular cutout; diaphragma bearing two sclerotized processes (costulae) near bases of valvae costa, bent at 90°, apex slightly swollen (Fig. 18); valve setose, sclerotized dorsally, membranous ventrally, Barth’s Organ robust, with numerous chevron-shaped parallel pleats, cucullus poorly defined with three narrow curved ridges, corona with weak, mesally-directed, curved setae. Aedeagus (Fig. 19) straight, abruptly flared out anteriorly, flattened, spoon shaped; vesica lightly sclerotized, with a patch of deciduous stellate (like a starfish) spicule-shaped cornuti; subbasal diverticulum with a nipple-shaped cornutus; apex lightly sclerotized, one large basal diverticulum with two subbasal lobes.

Adult female (Fig. 10). Similar to male except; antenna filiform without long setae; top of head yellow to orange; collar to disc of thorax yellow to orange. Forewing length = 16–18 mm, mean 17 mm, n = 9. *Genitalia* (Fig. 22). Papillae anales membranous, setose, hidden from view between sclerotized extensions of ninth abdominal segment; posterior apophyses slender; anterior apophyses slender; ductus bursae short, broad; corpus bursae round, with a single shark tooth-shaped signum; dorsal wall of corpus bursae sclerotized, forming a thumb-like extension, without bulbous terminus; sclerotized ventral wall with a pock-marked, finger-like extension appressed to surface of corpus bursae.

#### Remarks.

We make this a subspecies of *Elasmia mandela* because the color of the forewings is different from *Elasmia mandela mandela*, it is slightly smaller, and it is geographically separated from *Elasmia mandela*. The male and female genitalia, however, are indistinguishable from those of *Elasmia mandela mandela*. Some specimens from Oklahoma were previously misidentified as *Elasmia insularis*.

#### Distribution and biology.

In the U.S., *Elasmia mandela santaana* has been recorded from Texas and Oklahoma; its distribution in Mexico is unknown. A larval host (R. O. Kendall specimens in TAM) is *Unganadia speciosa* Endl. (Mexican buckeye) (Sapindaceae). The type locality was selected because the U.S. Fish and Wildlife Service will protect it into perpetuity.

**Figure:**
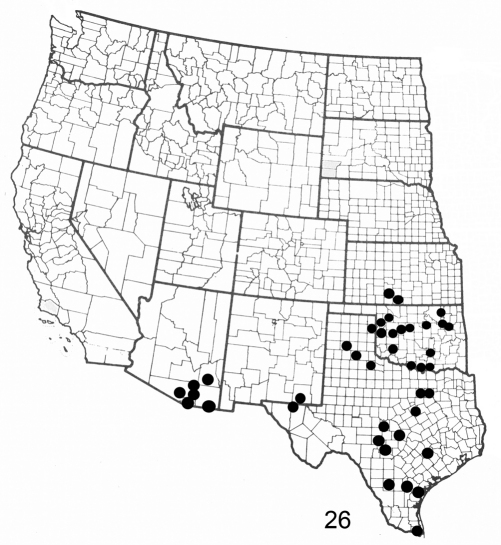
**Figure 26.** Distribution map for *Elasmia packardii* in United States.

**Figure:**
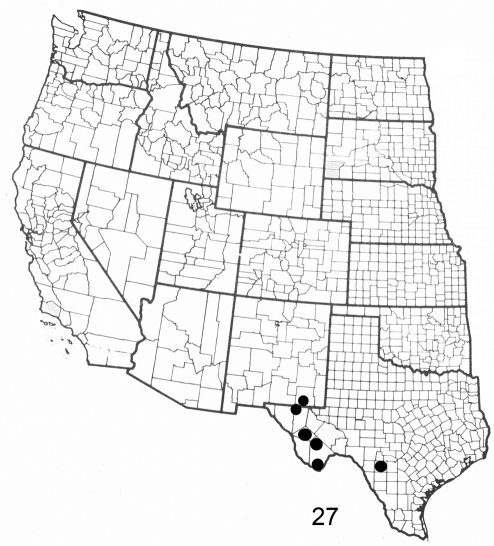
**Figure 27.** Distribution map for *Elasmia cave* in United States.

**Figure:**
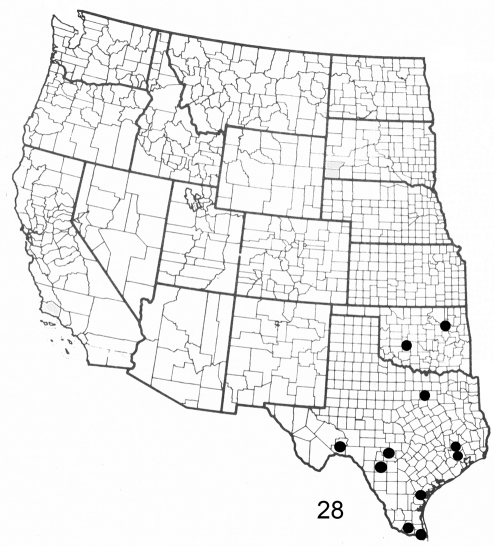
**Figure 28.** Distribution map for *Elasmia mandela santaana* in United States.

## Discussion

For all characters, except those we noted in the key and diagnoses, the species are closely similar in appearance.

The details of the shape of the costulae, in combination with the color and size of the adults, is important in defining the species. The costulae can be slightly variable within a species, thus all the characters should be consulted in making an identification.

The female genitalia of *Elasmia mandela mandela*, *Elasmia mandela santaana*, *Elasmia insularis*, *Elasmia packardii*, and *Elasmia cave* are nearly identical. The male genitalia of *Elasmia packardii* are distinct. The differences between the male genitalia of *Elasmia mandela*, *Elasmia insularis*, and *Elasmia cave* are more subtle; the most reliable character we found was the shape of the terminal portion of the costulae (Figs 12, 15, 18). The costulae of *Elasmia insularis* and *Elasmia mandela* are closely similar; the superficial appearance of the adults are different. The costulae of *Elasmia mandela santaana* and *Elasmia cave* are similar (see the key and Figs 15 and 18 for differences); most adults can be identified by external appearance, but a few specimens require examination of the male genitalia for positive identification.

The specimens from Carlsbad Caverns National Park were collected by Metzler as part of a 10-year study of the Lepidoptera of the Park initiated by the Park in 2006. This is the second in a series of papers (Metzler et al. 2010) detailing the moths of Carlsbad Caverns National Park.

## Supplementary Material

XML Treatment for Hippia

XML Treatment for Elasmia

XML Treatment for Elasmia insularis

XML Treatment for Elasmia packardii

XML Treatment for Elasmia cave

XML Treatment for Elasmia mandela

XML Treatment for Elasmia mandela santaana
